# Respiratory rhythm irregularity after carotid body denervation in rats

**DOI:** 10.1016/j.resp.2017.08.001

**Published:** 2017-12

**Authors:** Shahriar Sheikhbahaei, Alexander V. Gourine, Jeffrey C. Smith

**Affiliations:** a Cellular and Systems Neurobiology Section, National Institute of Neurological Disorders and Stroke (NINDS), National Institutes of Health (NIH), Bethesda, MD, USA; b Centre for Cardiovascular and Metabolic Neuroscience, Department of Neuroscience, Physiology, and Pharmacology, University College London, London WC1E 6BT, UK

**Keywords:** Breathing, Carotid body, Respiration, Irregular breathing, Sleep apnea, Sigh

## Abstract

Respiratory activity is controlled by inputs from the peripheral and central chemoreceptors. Since overactivity of the carotid bodies, the main peripheral chemoreceptors, is linked to the pathophysiology of disparate metabolic and cardiovascular diseases, carotid body denervation (CBD) has been proposed as a potential treatment. However, long-term effects of CBD on the respiratory rhythm and regularity of breathing remain unknown. Here, we show that five weeks after bilateral CBD in rats, the respiratory rhythm was slower and less regular. Ten weeks after bilateral CBD, the respiratory frequency was not different from the sham-operated group, but the regularity of the respiratory rhythm was still reduced. Increased frequency of randomly occurring apneas is likely to be responsible for the irregular breathing pattern after CBD. These results should be taken into consideration since any treatment that reduces the stability of the respiratory rhythm might exacerbate the cardio-respiratory instability and worsen the cardiovascular outcomes.

## Highlights

• CBD leads to a lower breathing frequency, which will be compensated over time.
• CBD causes an irregular breathing, which will not be compensated over time.
• CBD increases the rate of randomly occurring apnea, but has no effect on sigh frequency.

## 1 Introduction

Oxygenation of the arterial blood is continuously monitored by peripheral chemoreceptors located in the carotid and (in some species) aortic bodies (Heymans and Bouckaert, 1930, Heymans and Neil, 1958, O’Regan and Majcherczyk, 1982). These chemoreceptors detect changes in blood *P*O_2_ in a manner also dependent on blood *P*CO_2_/pH, and convey chemosensory information to the brainstem respiratory control networks (Bruce et al., 1982, Finley and Katz, 1992), which adjust the respiratory activity in accordance with prevailing physiological and behavioral needs.

Located bilaterally at the bifurcation of the common carotid arteries, carotid body chemoreceptors are believed to be the major site for respiratory oxygen sensing (Forster, 2003, Forster et al., 2000). When arterial *P*O_2_ decreases (e.g., during systemic hypoxia), the carotid bodies signal to the brainstem circuits, which increase the rate and depth of breathing (Heymans and Bouckaert, 1930) and also trigger adaptive increases in sympathetic activity (Persson et al., 1991, Persson and Kirchheim, 2013).

In addition to hypoxia, other stimuli, including hypercapnia (Andronikou et al., 1988, Forster et al., 2000), acidosis (Tan et al., 2007), hypoglycemia (Koyama et al., 2000, Pardal and López-Barneo, 2002), and mediators of inflammation (Ackland et al., 2013) have been shown to activate carotid body chemoreceptors. There is evidence that carotid body chemoreceptor hyperactivity may contribute to pathogenesis of certain metabolic and cardiovascular diseases, including hypertension (Abdala et al., 2012, Habeck, 1991, Trzebski et al., 1982), heart failure (Del Rio et al., 2013, Franchitto et al., 2010, Marcus et al., 2014, Ponikowski et al., 1997, Schultz et al., 2013), and insulin resistance (Ribeiro et al., 2013).

Recently, the efficacy of carotid body denervation as a potential treatment of sympathetically-mediated disease has been explored in animal models. In a rat model of obesity, carotid body denervation prevented development of hypertension and insulin resistance (Ribeiro et al., 2013). Carotid body denervation was also found to improve cardiac function in rat and rabbit models of heart failure (Del Rio et al., 2013, Marcus et al., 2014), and to reduce the degree of hypertension in spontaneously hypertensive rats (Abdala et al., 2012, McBryde et al., 2013). There is also human data suggesting that surgical removal of the carotid body may reduce the arterial blood pressure in some hypertensive patients (Nakayama, 1961, Narkiewicz et al., 2016, Winter and Whipp, 2004). Variable effects of uni- and bilateral carotid body resection in humans on respiratory activity, arterial blood pressure and heart rate have been reported (Timmers et al., 2003b). However, the available evidence comes from observations in patients in which carotid body resection was used as a treatment for certain underlying conditions including carotid body tumors (Timmers et al., 2003a) or chronic obstructive pulmonary disease (Whipp and Ward, 1992). The long-term effects of carotid body denervation on the respiratory rhythm stability remain unknown. In this study, we assessed the regularity of the respiratory rhythm in conscious adult rats five and ten weeks after bilateral carotid body ablation.

## 2 Methods

All the experiments were performed on male Sprague-Dawley rats in accordance with the European Commission Directive 2010/63/EU (European Convention for the Protection of Vertebrate Animals used for Experimental and Other Scientific Purposes), the UK Home Office (Scientific Procedures) Act (1986), and the National Institutes of Health Guide for the Care and Use of Laboratory Animals, with project approval from the respective Institutional Animal Care and Use Committees. Animals were housed in a temperature-controlled facility with a normal light-dark cycle (12 h:12 h, lights on at 7:00 A.M.). Tap water and laboratory rodent chow were provided *ad libitum*.

### 2.1 Ablation of the carotid body chemoreceptors

In young male rats (60–80 g, 3–4 weeks old), the carotid body chemoreceptors were ablated bilaterally as described in detail previously (Abdala et al., 2012, Angelova et al., 2015). Rats were anesthetized with intramuscular (i.m.) injection of a mixture of ketamine (60 mg kg^−1^) and medetomidine (250 μg kg^−1^). Using aseptic techniques, an anterior midline neck incision was made and the sternohyoid and sternocleidomastoid muscles were retracted. After exposing the carotid bifurcation, the occipital artery was carefully retracted, and the carotid body was visualized under a dissecting microscope. The carotid sinus nerve and its branches were cut, and the carotid bodies were removed. The same procedures were performed to expose the carotid bifurcation and both carotid bodies in the control group of rats, but the carotid sinus nerves and the carotid bodies were left intact (sham-operated animals). The neck incision was closed with absorbable suture and anesthesia was reversed with atipemazole (1 mg kg^−1^). No mortalities occurred after the surgery and the animals gained weight normally.

### 2.2 Recordings of the respiratory activity by whole body plethysmography

Five and ten weeks after bilateral carotid body ablation, whole-body plethysmography was used to record respiratory activity in unrestrained conscious rats, as described previously (Angelova et al., 2015, Trapp et al., 2011). The animals were maintained on a 12:12 h light–dark cycle. On the day of the experiment, the rat was placed in a Plexiglas recording chamber (∼1 L) that was flushed continuously with a humidified mixture of 79% N_2_ and 21% O_2_ (temperature 22–24 °C) at a rate of 1.2 L min^−1^. The animals were allowed to acclimatize to the chamber environment for at least 60 min before the recordings of respiratory-related plethysmographic signals were obtained. In order to limit the effect of the circadian rhythm on the respiratory activity, the recording sessions lasted for ∼2 h and took place between 11 A.M. and 3 P.M.

### 2.3 Data acquisition and analysis

Pressure changes in the plethysmography chamber were recorded using a Power1401 interface, and analyzed off-line using *Spike2* software (CED Limited, Cambridge, UK). The duration of the respiratory cycle (T_TOT_) was measured for each cycle after the rat had habituated to the recording chamber. After excluding movement and sniffing signal artifacts, the average T_TOT_ was calculated for an ∼2 h period of continuous recording and used to calculate the respiratory frequency (*f*_R_) presented as the number of respiratory cycles per minute. Variability of breathing was assessed by calculating the coefficient of variation (CV) and the irregularity score (IS) indices as described elsewhere (Telgkamp et al., 2002, Viemari et al., 2011). For each respiratory cycle, CV was determined by calculating the ratio of the standard deviation (SD) of the period of breathing cycles to the mean T_TOT_ and expressed as a percentage (CV = SD/mean T_TOT_ × 100). The IS was calculated by determining the value of (T_TOT*n*_ − T_TOT*n-1*_)/T_TOT*n-1*_ for the *n*^th^ respiratory cycle and also reported as a percentage. A lower irregularity score indicates a more regular respiratory rhythm. Poincaré plots of T_TOT_ for the n^th^ cycle versus T_TOT_ for n^th^ + 1 cycle were used to illustrate the temporal dispersion of T_TOT_. The frequency of sighs was also determined. A sigh was defined as a high-amplitude inspiratory breath that started near the peak of the normal inspiration and was at least 100% larger in amplitude than the mean amplitude of five breaths proceeding each sigh (Cherniack et al., 1981). Sighs were typically followed by a period of post-sigh apnea. Sigh frequency is expressed as the number of sighs per hour. Periods of apnea were also identified by the absence of respiratory activity over a period of at least three complete respiratory cycles (i.e., ≥3xT_TOT_). The apnea index was expressed as the number of apneas per hour. The data were compared by Mann-Whitney U by ranks and reported as means ± SEM. Differences between experimental groups with p < 0.05 were considered to be statistically significant.

### 2.4 Assessment of sleep efficiency

To confirm successful ablation of the carotid bodies, the animals were exposed to hypoxia while asleep and sleep efficiency was assessed as described in detail previously (Angelova et al., 2015). Briefly, an investigator who was unaware of the nature of the experimental groups used established behavioral criteria (Gramsbergen et al., 1970) to calculate sleep efficiency score (SES) before, during, and after the hypoxic challenge. SES was defined as the percentage of the time an animal spent is quiet sleep (QS) and active sleep (AS) according to the following formula: SES = 100x(QS + AS)/(QS + AS + W + IN), where W was the total time the rat was awake. When it was difficult to establish the state definitely, this time period was designated as indeterminate (IN).

## 3 Results

### 3.1 Verification of successful ablation of the carotid bodies

**Fig. 1:**
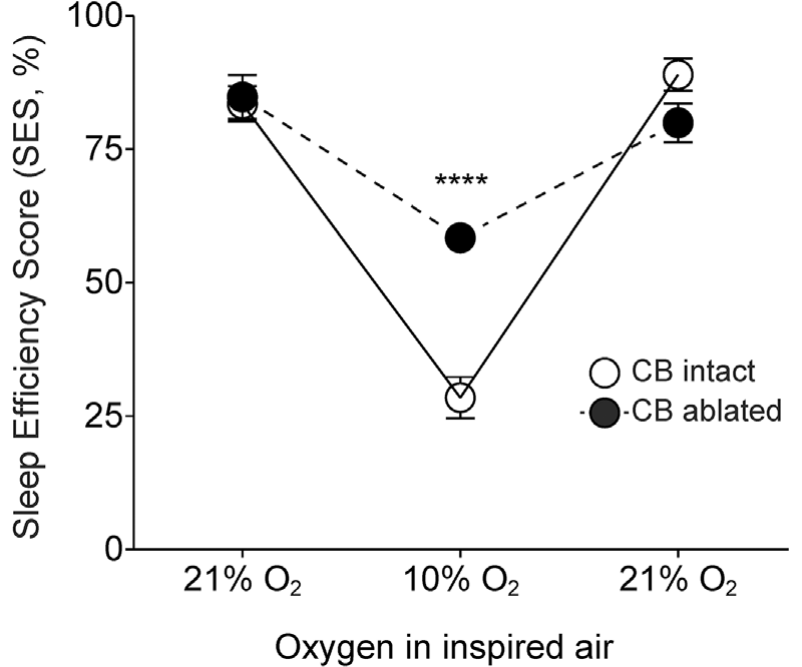
Hypoxia-induced arousal responses in rats following bilateral ablation of the carotid bodies. Summary data illustrating changes in SES induced by hypoxia in the carotid body ablated and sham-operated animals (10 weeks after CB ablation, n = 8 per group).

Conscious rats (as well as other mammals, but not humans) with denervated peripheral chemoreceptors display partial or almost complete recovery of the ventilatory response to hypoxia (Angelova et al., 2015, Bisgard et al., 1980, Davenport and Brewer, 1947, Miller and Tenney, 1975, Olson et al., 1988; for recent review see also Gourine and Funk, 2017). Therefore, successful ablation of the carotid bodies cannot be reliably verified by measuring the hypoxic ventilatory response. However, peripheral oxygen chemoreceptors are believed to play a key role in triggering a hypoxia-induced arousal response (Bowes et al., 1981, Miller and Tenney, 1975). Therefore, in the absence of the carotid body input, hypoxic challenge should minimally affect sleep efficiency. We first assessed the effect of a brief period (10 min) of hypoxia (10% O_2_ in the inspired air) on sleep efficiency in sham-operated and carotid body-ablated rats by calculating SES before, during, and after the hypoxic challenge. SES was not different in the carotid body-denervated and sham-operated animals under normoxic/normocapnic conditions (84 ± 4% vs. 85 ± 3%, p = 0.6; Fig. 1). When the animals were exposed to 10% O_2_, SES was found to be significantly higher in rats with ablated carotid bodies compared to sham-operated animals (58 ± 1% vs. 29 ± 3%, p < 0.0001; Fig. 1). These data confirmed that in rats, afferent inputs from the carotid body chemoreceptors remain absent/impaired ten weeks after denervation.

### 3.2 Resting respiratory frequency in conditions of chronic carotid body ablation in rats

**Fig. 2:**
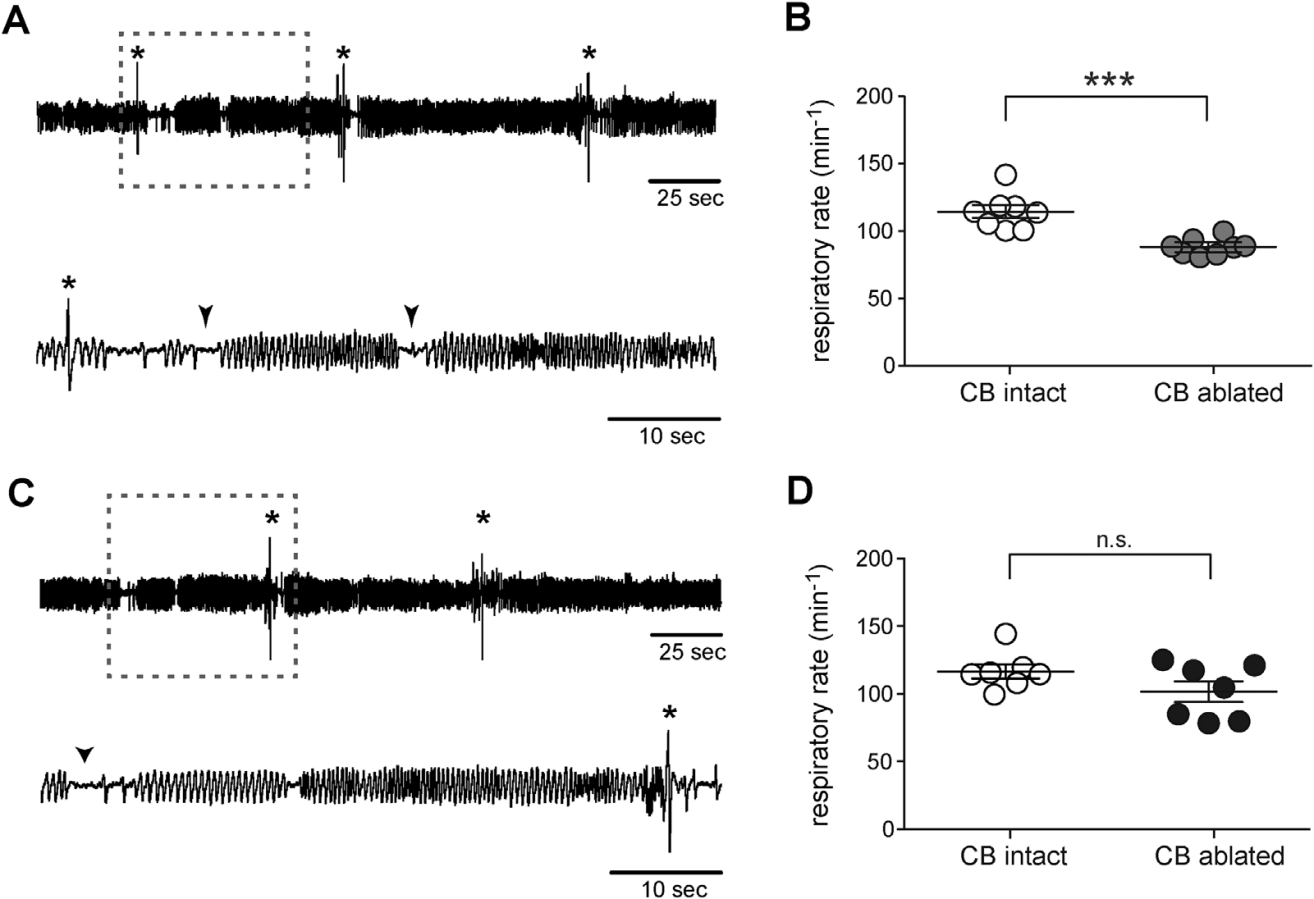
Resting breathing patterns after carotid body ablation. (A) Representative time-condensed and expanded traces (for the period indicated by the dashed box) illustrating resting breathing pattern in a conscious rat five weeks after bilateral carotid body ablation, showing sighs (marked by *) and randomly occurring apneas (marked by arrow heads). (B) Summary data showing 23 ± 3% decrease in the mean resting breathing rate (*f*_R_) in conscious rats five weeks after bilateral carotid body ablation when compared to the sham-operated rats. (C) Representative time-condensed and expanded traces illustrating resting breathing pattern in a conscious rat ten weeks after bilateral carotid body ablation. (D) Summary data showing no difference in the mean resting *f*_R_ in conscious rats ten weeks after bilateral carotid body ablation compared to the sham-operated animals. Sighs (*) and random apneas (arrow heads) are indicated.

During the light phase of the 24 h cycle, when the animals spent most of the time asleep, the resting rate of breathing (*f*_R_) in the carotid body-ablated rats five weeks after the surgery, was ∼25% lower (88 ± 2 breaths min^−1^) compared to sham-operated animals (115 ± 5 breaths min^−1^, n = 8, p < 0.001) (Fig. 2A&B). Respiratory frequency in the carotid body-ablated rats was not different from that in sham-operated rats ten weeks after carotid body ablation (102 ± 8 vs. 117 ± 5 breaths min^−1^, n = 7, p = 0.5; Fig. 2C&D).

### 3.3 Regularity of breathing in conditions of chronic carotid body ablation in rats

**Fig. 3:**
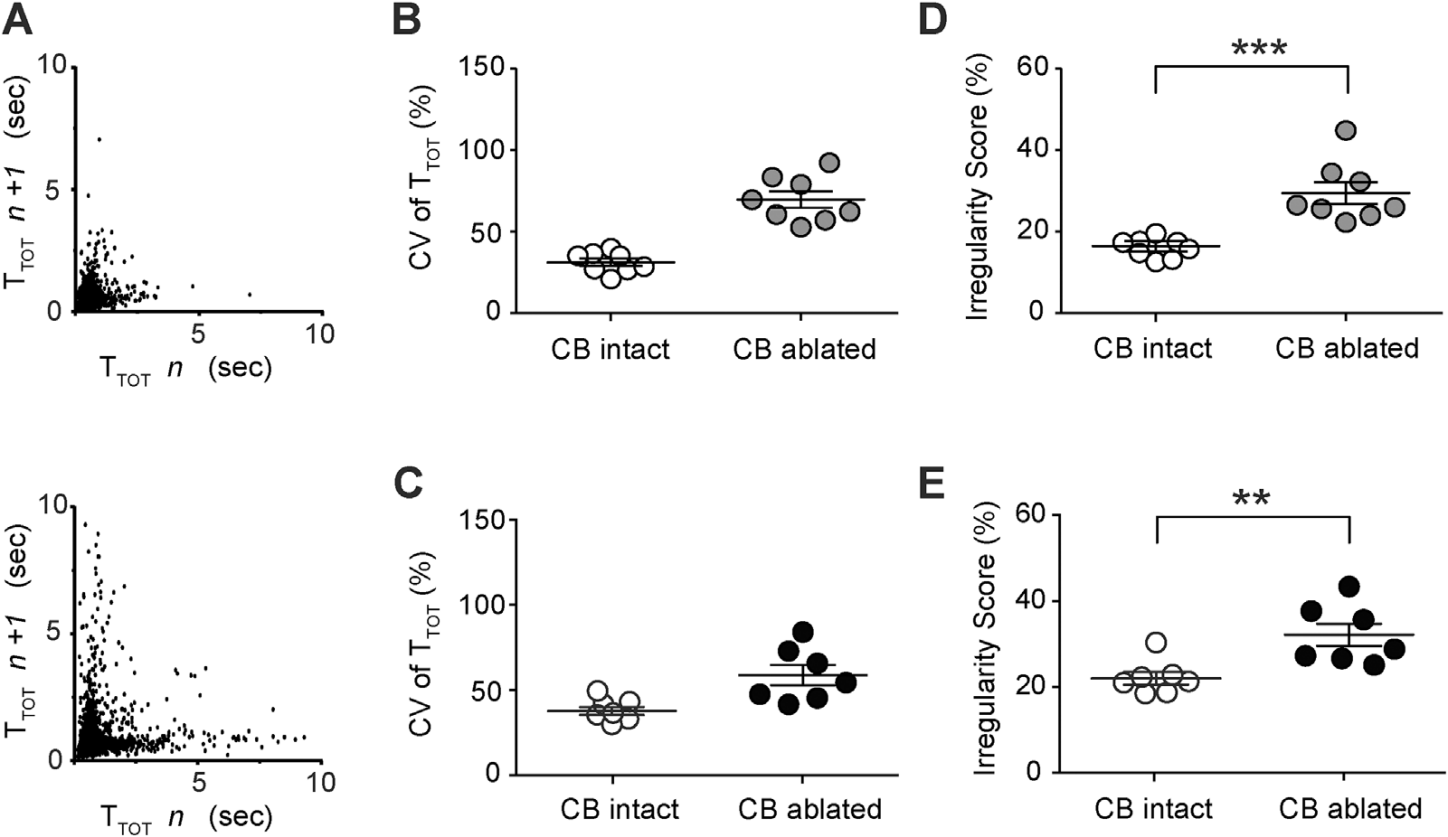
Irregular breathing pattern in conditions of chronic carotid body ablation. (A) Poincaré plots of the respiratory cycle duration (T_TOT_) for n^th^ cycle versus T_TOT_ for the n^th^ + 1 cycle in a sham-operated rat (top) and in a rat five weeks after carotid bodies ablation (bottom). Summary data showing that the coefficient of variation (CV) of T_TOT_ was higher in carotid body ablated rats five (B) and ten (C) weeks after surgery when compared to sham-operated rats. (D) Summary data showing that the breathing Irregularity Score (IS) was higher in carotid body ablated rats vs. sham-operated animals five weeks after surgery. (E) Summary data showing that IS remained significantly higher ten weeks after bilateral ablation of the carotid bodies when compared to sham-operated rats.

Higher cycle-to-cycle dispersion in T_TOT_ in conditions of peripheral chemodenervation is clearly evident when illustrated using Poincaré plots (Fig. 3A). To determine the effect of carotid body ablation on the regularity of breathing, two parameters were assessed: coefficient of variation (CV) of the breath-to-breath time (T_TOT_) and irregularity score (IS). Five weeks after peripheral chemodenervation, CV was higher in carotid body-ablated rats compared to sham-operated animals (70 ± 5% vs. 31 ± 3%, n = 8; Fig. 3B). IS was also higher in carotid body-ablated rats (29 ± 3% vs. 16 ± 1%, p < 0.001; Fig. 3D). Both CV and IS were also higher when assessed ten weeks after carotid body ablation (Fig. 3C, E).

### 3.4 Sigh frequency and apnea index in conditions of chronic carotid body ablation in rats

**Fig. 4:**
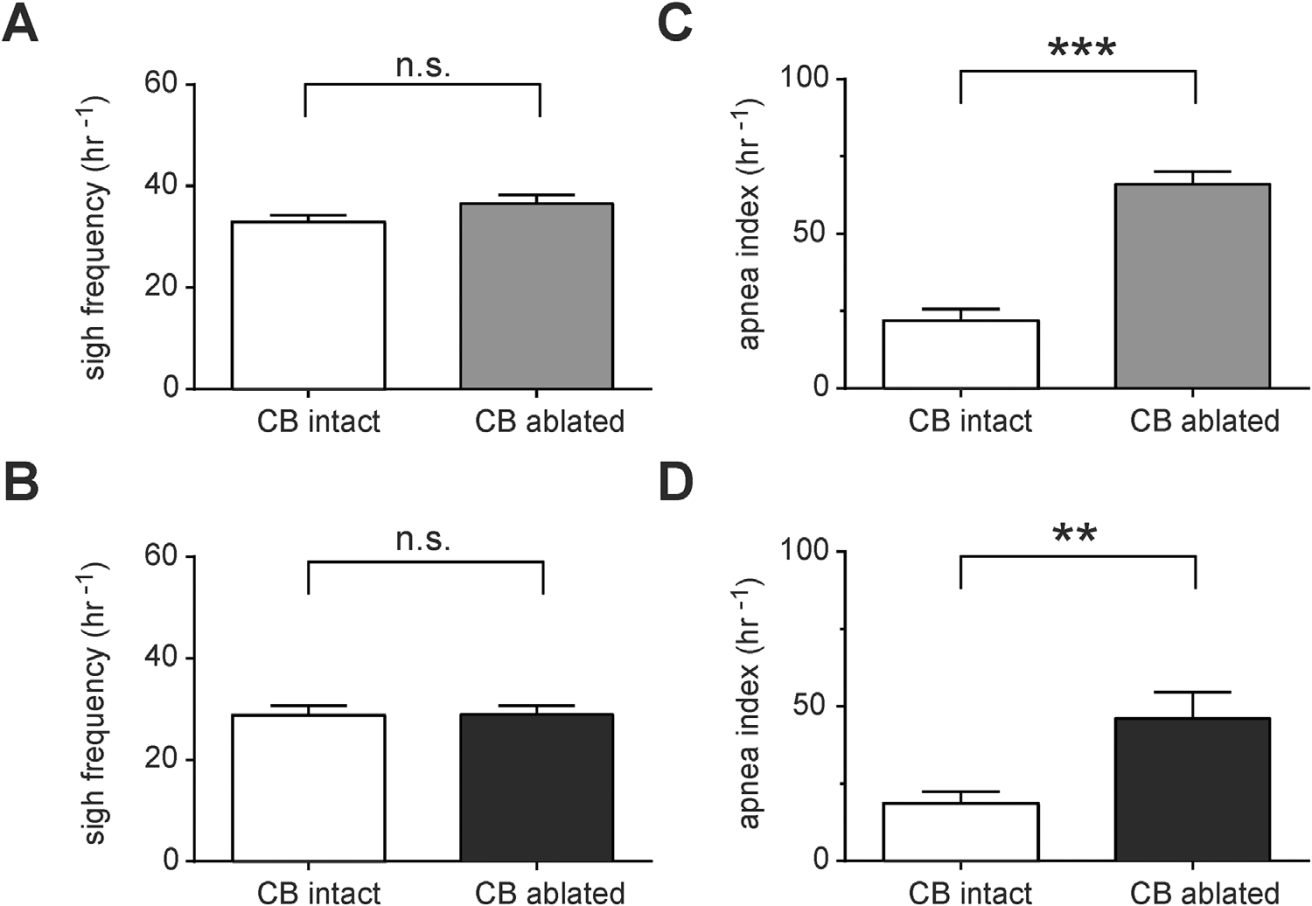
*S*igh frequency and apnea index in conditions of chronic carotid body ablation. When compared to sham-operated rats, sigh frequencies were not different five (A) or ten (B) weeks after carotid body ablation. (C) Summary data showing that apnea index (random events per hour) was significantly higher in rats five weeks after carotid body ablation compared to sham-operated rats. (D) Summary data showing that apnea index remained higher in carotid body ablated rats ten weeks after ablation when compared to sham-operated rats.

Sighs and periods of apnea could potentially contribute to breathing irregularity. Sigh and the post-sigh pause of inspiratory activity are believed to be generated within inspiratory rhythm-generating circuits of the preBötzinger complex (preBötC) (Lieske et al., 2000, Ramirez et al., 2013), which receive excitatory afferent inputs originating from the carotid bodies. Apneas may also occur in the absence of the carotid body input. Sigh frequencies were not different between the experimental groups five (37 ± 2 in sham-operated rats vs. 33 ± 2 h^−1^ in CB ablated rats; p = 0.6; Fig. 4A) and ten (29 ± 3 in sham-operated rats vs. 29 ± 4 h^−1^ in CB ablated rats; p = 0.8; Fig. 4B) weeks after the carotid body ablation. After five weeks, the frequency of randomly occurring apneas was significantly higher in carotid body-ablated rats compared to sham-operated animals (64 ± 2% vs. 22 ± 4%, n = 8, p < 0.001; Fig. 4C). Similarly, the apnea index was higher ten weeks after bilateral ablation of the carotid bodies (46 ± 8% vs. 19 ± 4% in sham-operated animals; p = 0.01; Fig. 4D).

## 4 Discussion

This study determined the role of afferent inputs from the carotid body chemoreceptors in maintaining regularity of breathing in conscious rats. We hypothesized that respiratory network activity would display higher variability when the afferent inputs from this key peripheral respiratory chemoreceptor site are removed experimentally. Indeed, when regularity of breathing was assessed by two different independent criteria (IS and CV of T_TOT_), ablation of the carotid bodies was found to be associated with a slower and less regular respiratory rhythm five weeks after the denervation surgery (regularity of breathing was assessed during the light-phase of the 24 h cycle when the animals spent most of the time asleep). Ten weeks after the carotid body ablation, respiratory frequency was not different from that displayed by sham-operated rats, but regularity of the respiratory rhythm was still reduced. Moreover, ablation of the carotid bodies had no effect on sigh frequency five and ten weeks after the surgery, indicating that peripheral chemoreceptor afferent inputs are not essential for generation of the inspiratory sighs (Lieske et al., 2000, Li et al., 2016). Importantly, five weeks after carotid body ablation, the apnea index was increased by nearly two-fold, and remained elevated ten weeks after peripheral chemoreceptor denervation due to a higher frequency of randomly occurring apneas (Fig. 4).

Respiratory activity in mammals is generated by brainstem neural circuits, including the inspiratory rhythm generating circuits in the medullary preBötC (Smith et al., 1991). Brainstem respiratory networks receive afferent inputs from central and peripheral respiratory chemoreceptors to adjust lung ventilation in accord with prevailing metabolic and behavioral needs (Feldman et al., 2003, Funk, 2013). Results obtained in this study suggest that afferent activity of the carotid body chemoreceptors provides an important tonic input that contributes to the stability of the respiratory rhythm. Similarly, denervation of the arterial baroreceptors is well known to increase the variability of systemic arterial blood pressure (Alper et al., 1987, Sved et al., 1997).

Recently, carotid body denervation has been put forward as a potential therapy/treatment of certain disease states characterized by high sympathetic activity which is generally considered to have a detrimental effect, contributing to the development and progression of certain cardiovascular pathologies (Paton et al., 2013). Indeed, results of several experimental studies demonstrated a clear beneficial effect of carotid body denervation in animal models of hypertension and heart failure (Abdala et al., 2012, Del Rio et al., 2013, Marcus et al., 2014, Paton et al., 2013). The first clinical reports are now published describing the effects of unilateral carotid body denervation in heart failure patients and patients with drug-resistant hypertension (Narkiewicz et al., 2016, Niewinski et al., 2017, Niewiński et al., 2013). In contrast to the results of the animal experiments, no effect of carotid body denervation on blood pressure was observed in patients with drug-resistant hypertension (Narkiewicz et al., 2016). In addition, the chronic effect of peripheral chemoreceptor denervation on respiratory rhythm stability under normal physiological conditions has not been reported. This appears to be important since a sleep-disordered breathing pattern is frequent in heart failure: 27% of heart failure patients have obstructive sleep apnea and 38% have central sleep apnea (Bradley and Floras, 2003a, Bradley and Floras, 2003b). Sleep-disordered breathing is strongly associated and believed to contribute to sympathetic over-activity and progression of cardiovascular disease (Chouchou et al., 2013). Previous studies in rat models of heart failure reported that carotid body denervation decreases variability of breathing (Del Rio et al., 2013). In contrast, when we measured variability of breathing five and ten weeks after carotid body ablation, it was found to be higher in rats that underwent bilateral ablation of carotid bodies when compared to sham-operated rats. These differences might be due to the different protocols for carotid body denervation [excision of carotid bodies in young (this study) vs. adult animals (Del Rio et al., 2013)] and differences in data analysis [measuring variability of breathing in periods of ∼ 120 min (this study) vs. ∼ 5 min (Del Rio et al., 2013)]. Nevertheless, in humans and animal models, denervation of the carotid bodies results in higher resting *P*CO_2_ (during sleep *P*CO_2_ may further increase), and it is proposed that increases in *P*CO_2_ may lead to unstable breathing (Dempsey et al., 2004). Indeed, it was reported that surgical removal of both carotid bodies made patients susceptible to the development of irregular breathing patterns (Dahan et al., 2007). The results obtained in this study should be taken into consideration since any treatment that reduces stability of the respiratory rhythm over time might detrimentally exaggerate the cardio-respiratory instability and worsen the cardiovascular outcomes.

## Conflict of interest

The authors declare no competing financial interests.

## Funding

This work was supported by The Wellcome Trust (A.V.G.) and by the Intramural Research Program of the NIH, NINDS. A.V.G is a Wellcome Trust Senior Research Fellow (Refs: 095064 and 200893). S.S. is an NIH-UCL GPP Fellow.

## References

[bib0005] Abdala A.P., McBryde F.D., Marina N., Hendy E.B., Engelman Z.J., Fudim M., Sobotka P.A., Gourine A.V., Paton J.F.R. (2012). Hypertension is critically dependent on the carotid body input in the spontaneously hypertensive rat. J. Physiol. (Lond.).

[bib0010] Ackland G.L., Kazymov V., Marina N., Singer M., Gourine A.V. (2013). Peripheral neural detection of danger-associated and pathogen-associated molecular patterns. Crit. Care Med..

[bib0015] Alper R.H., Jacob H.J., Brody M.J. (1987). Regulation of arterial pressure liability in rats with chronic sinoaortic deafferentation. Am. J. Physiol..

[bib0020] Andronikou S., Shirahata M., Mokashi A., Lahiri S. (1988). Carotid body chemoreceptor and ventilatory responses to sustained hypoxia and hypercapnia in the cat. Respir. Physiol..

[bib0025] Angelova P.R., Kasymov V., Christie I., Sheikhbahaei S., Turovsky E., Marina N., Korsak A., Zwicker J., Teschemacher A.G., Ackland G.L., Funk G.D., Kasparov S., Abramov A.Y., Gourine A.V. (2015). Functional oxygen sensitivity of astrocytes. J. Neurosci..

[bib0030] Bisgard G.E., Forster H.V., Klein J.P. (1980). Recovery of peripheral chemoreceptor function after denervation in ponies. J. Appl. Physiol..

[bib0035] Bowes G., Townsend E.R., Kozar L.F., Bromley S.M., Phillipson E.A. (1981). Effect of carotid body denervation on arousal response to hypoxia in sleeping dogs. J. Appl. Physiol..

[bib0040] Bradley T.D., Floras J.S. (2003). Sleep apnea and heart failure: part I: obstructive sleep apnea. Circulation.

[bib0045] Bradley T.D., Floras J.S. (2003). Sleep apnea and heart failure: part II: central sleep apnea. Circulation.

[bib0050] Bruce E.N., Mitra J., Cherniack N.S. (1982). Central and peripheral chemoreceptor inputs to phrenic and hypoglossal motoneurons. J. Appl. Physiol..

[bib0055] Cherniack N.S., Euler von C., Głogowska M., Homma I. (1981). Characteristics and rate of occurrence of spontaneous and provoked augmented breaths. Acta Physiol. Scand..

[bib0060] Chouchou F., Pichot V., Pépin J.L., Tamisier R., Celle S., Maudoux D., Garcin A., Lévy P., Barthélémy J.C., Roche F., PROOF Study Group (2013). Sympathetic overactivity due to sleep fragmentation is associated with elevated diurnal systolic blood pressure in healthy elderly subjects: the PROOF-SYNAPSE study. Eur. Heart J..

[bib0065] Dahan A., Nieuwenhuijs D., Teppema L. (2007). Plasticity of central chemoreceptors: effect of bilateral carotid body resection on central CO2 sensitivity. PLoS Med..

[bib0070] Davenport H.W., Brewer G. (1947). The respiratory responses to anoxemia of unanesthetized dogs with chronically denervated aortic and carotid chemoreceptors and their causes. Am. J. Physiol..

[bib0075] Del Rio R., Marcus N.J., Schultz H.D. (2013). Carotid chemoreceptor ablation improves survival in heart failure: rescuing autonomic control of cardiorespiratory function. J. Am. Coll. Cardiol..

[bib0080] Dempsey J.A., Smith C.A., Przybylowski T., Chenuel B., Xie A., Nakayama H., Skatrud J.B. (2004). The ventilatory responsiveness to CO(2) below eupnoea as a determinant of ventilatory stability in sleep. J. Physiol. (Lond.).

[bib0085] Feldman J.L., Mitchell G.S., Nattie E.E. (2003). Breathing: rhythmicity, plasticity, chemosensitivity. Annu. Rev. Neurosci..

[bib0090] Finley J.C., Katz D.M. (1992). The central organization of carotid body afferent projections to the brainstem of the rat. Brain Res..

[bib0095] Forster H.V., Pan L.G., Lowry T.F., Serra A., Wenninger J., Martino P. (2000). Important role of carotid chemoreceptor afferents in control of breathing of adult and neonatal mammals. Respir. Physiol..

[bib0100] Forster H.V. (2003). Plasticity in the control of breathing following sensory denervation. J. Appl. Physiol..

[bib0105] Franchitto N., Despas F., Labrunée M., Roncalli J., Boveda S., Galinier M., Senard J.-M., Pathak A. (2010). Tonic chemoreflex activation contributes to increased sympathetic nerve activity in heart failure-related anemia. Hypertension.

[bib0110] Funk G.D. (2013). Neuromodulation: purinergic signaling in respiratory control. Compr. Physiol..

[bib0115] Gourine A.V., Funk G.D. (2017). On the existence of a central respiratory oxygen sensor. J. Appl. Physiol..

[bib0120] Gramsbergen A., Schwartze P., Prechtl H.F. (1970). The postnatal development of behavioral states in the rat. Dev. Psychobiol..

[bib0125] Habeck J.-O. (1991). Peripheral arterial chemoreceptors and hypertension. J. Auton. Nerv. Syst..

[bib0130] Heymans C., Bouckaert J.J. (1930). Sinus caroticus and respiratory reflexes. J. Physiol. (Lond.).

[bib0135] Heymans C., Neil E. (1958). Reflexogenic areas of the cardiovascular system. Br. J. Surg..

[bib0140] Koyama Y., Coker R.H., Stone E.E., Lacy D.B., Jabbour K., Williams P.E., Wasserman D.H. (2000). Evidence that carotid bodies play an important role in glucoregulation in vivo. Diabetes.

[bib0145] Li P., Janczewski W.A., Yackle K., Kam K., Pagliardini S., Krasnow M.A., Feldman J.L. (2016). The peptidergic control circuit for sighing. Nature.

[bib0150] Lieske S.P., Thoby-Brisson M., Telgkamp P., Ramirez J.M. (2000). Reconfiguration of the neural network controlling multiple breathing patterns: eupnea, sighs and gasps. Nat. Neurosci..

[bib0155] Marcus N.J., Del Rio R., Schultz E.P., Xia X.-H., Schultz H.D. (2014). Carotid body denervation improves autonomic and cardiac function and attenuates disordered breathing in congestive heart failure. J. Physiol. (Lond.).

[bib0160] McBryde F.D., Abdala A.P., Hendy E.B., Pijacka W., Marvar P., Moraes D.J.A., Sobotka P.A., Paton J.F.R. (2013). The carotid body as a putative therapeutic target for the treatment of neurogenic hypertension. Nat. Commun..

[bib0165] Miller M.J., Tenney S.M. (1975). Hypoxia-induced tachypnea in carotid-deafferented cats. Respir. Physiol..

[bib0170] Nakayama K. (1961). Surgical removal of the carotid body for bronchial asthma. Chest.

[bib0175] Narkiewicz K., Ratcliffe L.E.K., Hart E.C., Briant L.J.B., Chrostowska M., Wolf J., Szyndler A., Hering D., Abdala A.P., Manghat N., Burchell A.E., Durant C., Lobo M.D., Sobotka P.A., Patel N.K., Leiter J.C., Engelman Z.J., Nightingale A.K., Paton J.F.R. (2016). Unilateral carotid body resection in resistant hypertension: a safety and feasibility trial. JACC Basic Transl. Sci..

[bib0180] Niewiński P., Janczak D., Rucinski A., Jazwiec P., Sobotka P.A., Engelman Z.J., Fudim M., Tubek S., Jankowska E.A., Banasiak W., Hart E.C.J., Paton J.F.R., Ponikowski P. (2013). Carotid body removal for treatment of chronic systolic heart failure. Int. J. Cardiol..

[bib0185] Niewinski P., Janczak D., Rucinski A., Tubek S., Engelman Z.J., Piesiak P., Jazwiec P., Banasiak W., Fudim M., Sobotka P.A., Javaheri S., Hart E.C.J., Paton J.F.R., Ponikowski P. (2017). Carotid body resection for sympathetic modulation in systolic heart failure: results from first-in-man study. Eur. J. Heart Fail..

[bib0190] O’Regan R.G., Majcherczyk S. (1982). Role of peripheral chemoreceptors and central chemosensitivity in the regulation of respiration and circulation. J. Exp. Biol..

[bib0195] Olson E.B., Vidruk E.H., Dempsey J.A. (1988). Carotid body excision significantly ventilatory control in awake rats changes. J. Appl. Physiol..

[bib0200] Pardal R., López-Barneo J. (2002). Low glucose-sensing cells in the carotid body. Nat. Neurosci..

[bib0205] Paton J.F.R., Sobotka P.A., Fudim M., Engelman Z.J., Hart E.C.J., McBryde F.D., Abdala A.P., Marina N., Gourine A.V., Lobo M., Patel N., Burchell A., Ratcliffe L., Nightingale A. (2013). The carotid body as a therapeutic target for the treatment of sympathetically mediated diseases. Hypertension.

[bib0210] Persson P.B., Kirchheim H.R. (1991). Baroreceptor Reflexes: Integrative Functions and Clinical Aspects.

[bib0215] Persson P.B., Ehmke H., Kirchheim H.R. (1991). Blood pressure control in arterial- and cardiopulmonary receptor denervated dogs. Acta Physiol. Scand..

[bib0220] Ponikowski P., Chua T.P., Piepoli M., Ondusova D., Webb-Peploe K., Harrington D., Anker S.D., Volterrani M., Colombo R., Mazzuero G., Giordano A., Coats A.J. (1997). Augmented peripheral chemosensitivity as a potential input to baroreflex impairment and autonomic imbalance in chronic heart failure. Circulation.

[bib0225] Ramirez J.-M., Garcia A.J., Anderson T.M., Koschnitzky J.E., Peng Y.-J., Kumar G.K., Prabhakar N.R. (2013). Central and peripheral factors contributing to obstructive sleep apneas. Respir. Physiol. Neurobiol..

[bib0230] Ribeiro M.J., Sacramento J.F., Gonzalez C., Guarino M.P., Monteiro E.C., Conde S.V. (2013). Carotid body denervation prevents the development of insulin resistance and hypertension induced by hypercaloric diets. Diabetes.

[bib0235] Schultz H.D., Marcus N.J., Del Rio R. (2013). Role of the carotid body in the pathophysiology of heart failure. Curr. Hypertens. Rep..

[bib0240] Smith J.C., Ellenberger H.H., Ballanyi K., Richter D.W., Feldman J.L. (1991). Pre-Bötzinger complex: a brainstem region that may generate respiratory rhythm in mammals. Science.

[bib0245] Sved A.F., Schreihofer A.M., Kost C.K. (1997). Blood pressure regulation in baroreceptor-denervated rats. Clin. Exp. Pharmacol. Physiol..

[bib0250] Tan Z.-Y., Lu Y., Whiteis C.A., Benson C.J., Chapleau M.W., Abboud F.M. (2007). Acid-sensing ion channels contribute to transduction of extracellular acidosis in rat carotid body glomus cells. Circ. Res..

[bib0255] Telgkamp P., Cao Y.Q., Basbaum A.I., Ramirez J.M. (2002). Long-term deprivation of substance P in PPT-a mutant mice alters the anoxic response of the isolated respiratory network. J. Neurophysiol..

[bib0260] Timmers H.J.L.M., Karemaker J.M., Wieling W., Marres H.A.M., Folgering H.T.M., Lenders J.W.M. (2003). Baroreflex and chemoreflex function after bilateral carotid body tumor resection. J. Hypertens..

[bib0265] Timmers H.J.L.M., Wieling W., Karemaker J.M., Lenders J.W.M. (2003). Denervation of carotid baro- and chemoreceptors in humans. J. Physiol. (Lond.).

[bib0270] Trapp S., Tucker S.J., Gourine A.V. (2011). Respiratory responses to hypercapnia and hypoxia in mice with genetic ablation of Kir5.1 (Kcnj16). Exp. Physiol..

[bib0275] Trzebski A., Tafil M., Zoltowski M., Przybylski J. (1982). Increased sensitivity of the arterial chemoreceptor drive in young men with mild hypertension. Cardiovasc. Res..

[bib0280] Viemari J.-C., Garcia A.J., Doi A., Ramirez J.-M. (2011). Activation of alpha-2 noradrenergic receptors is critical for the generation of fictive eupnea and fictive gasping inspiratory activities in mammals in vitro. Eur. J. Neurosci..

[bib0285] Whipp B.J., Ward S.A. (1992). Physiologic changes following bilateral carotid-body resection in patients with chronic obstructive pulmonary disease. Chest.

[bib0290] Winter B., Whipp B.J. (2004). Immediate effects of bilateral carotid body resection on total respiratory resistance and compliance in humans. Adv. Exp. Med. Biol..

